# Supplementary material for: Quantitative phase microscopies: accuracy comparison
Source: Light Sci Appl. 2024 Oct 11;13:288. doi: 10.1038/s41377-024-01619-7 (PMC11470049; doi:10.1038/s41377-024-01619-7)
Supplement: Supplementary file 1 — Supplementary material file [file 41377_2024_1619_MOESM1_ESM.pdf]

SUPPLEMENTARY INFORMATION FOR

QUANTITATIVE PHASE MICROSCOPIES: ACCURACY COMPARISON

PATRICK C. CHAUMET[1], PIERRE BON[2], GUILLAUME MAIRE[1], ANNE SENTENAC[1], AND GUILLAUME BAFFOU[1,3]

[1] Institut Fresnel, CNRS, Aix Marseille Univ, Centrale Med, Marseille, France
[2] Université de Limoges, CNRS, XLIM, UMR 7252, F-87000 Limoges, France
[3] Neurotechnology Center, Department of Biological Sciences, Columbia University, New York, NY 10027, USA

1. PATTERN OF THE LED ARRAY IN DPC

Figure S1 represents the distribution of the 192 point-like sources used in DPC simulations. The array is cut in 4 quadrants. Contiguous quadrants are turned on two by two to successively apply top, right, left and bottom illuminations.

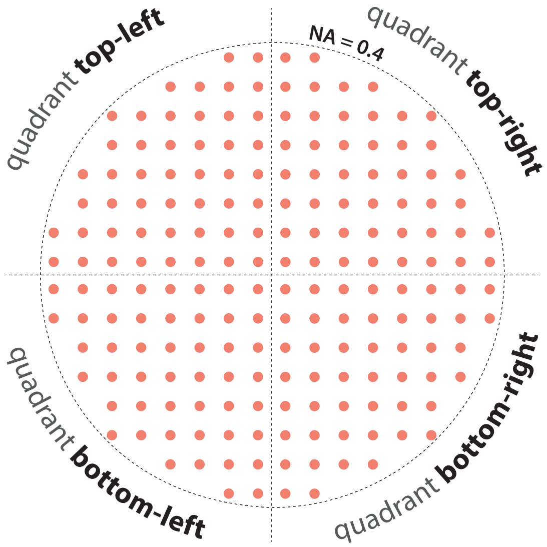

Fig. S1. Geometry of the point-like sources in the front focal plane of the condensor used in DPC simulations.

2. RESULTS FOR PSI

Figure S2 complements Figures 6 and 7 of the main manuscript with the results on PSI microscopy.

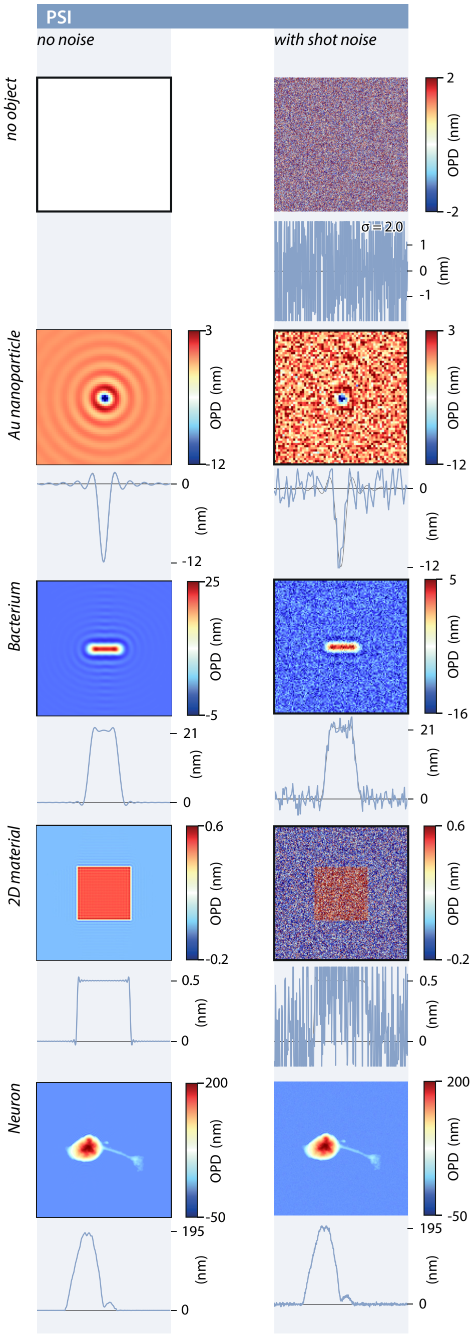

Fig. S2. 9 numerical simulations of OPD images of 4 different objects, acquired using PSI microscopy, on the same microscope. Left column: simulations without noise. Right column: simulations with noise. Each row corresponds to an object. For each image, an horizontal crosscut passing through the center of the image is displayed below. The grey lines in the background indicate the theoretical profiles.

3. ONLINE INFORMATION

A. Codes on Github / Gitlab

The IFDDA package is available at `https://gitlab.com/ifdda/if-ddam`.

The codes related to the modelisation of the microscopes is available at `https://github.com/baffou/PhaseLAB`, in the `src/functions/+IF` folder.

The `QPMcomparison.m` file is the main script that was used to run all the simulations. The other important file is `wavecomputation.m`, called by `QPMcomparison.m`.

B. Data on Zenodo

This repository `https://zenodo.org/doi/10.5281/zenodo.10888480` contains the raw data displayed in the article.

- Folder `theo` contains the data of Fig. 5
- Folder `nonoise` contains the data of Fig. 6
- Folder `noisy` contains the data of Fig. 7
- Folder `230728_CGMdistance` contains data of Fig. 9
- Folder `230728_DPMdiameter` contains data of Fig. 11a,b
- Folder `230728_DPMdiameter_NA` contains data of Fig. 11c,d
- Folder `230728_FPMdiameter` contains data of Fig. 13a,
- Folder `230728_FPMdiameter_NA` contains data of Fig. 13c,d
- Folder `230728_TIEzShift_NP` contains data of Fig. 14a,c.
- Folder `230728_TIEzShift_cell` contains data of Fig. 14b,d.

4. METRICS ON TRUENESS AND PRECISION

Figure 8 plots quantitative results on measurement trueness and precision. Below are the values plotted in the graphs of Fig 8.

Imaginary part of the polarizability of the NP [10-3 /µm3]

with noise

DHM	CGM	DPM	DPC	PSI	FPM	SLIM	TIE
3.649	3.4543	4.3683	3.0515	4.3132	3.5937	3.2556	3.5582
3.5551	3.2692	3.5698	3.0084	3.796	3.5742	3.403	3.5586
3.2797	3.3444	4.2852	2.9013	3.8849	3.5819	3.2897	3.5718
3.7078	3.5087	3.7709	2.8095	3.3767	3.6345	3.4144	3.559
3.4667	3.3946	3.2519	3.0525	3.995	3.4841	3.4455	3.5868
3.3829	3.3405	4.37	2.9691	3.4461	3.6774	3.3871	3.5548
3.6626	3.3352	3.8798	2.9182	3.7872	3.5069	3.4605	3.5636
3.4021	3.4274	3.3546	3.0412	3.7114	3.4902	3.4772	3.5666
3.606	3.3658	4.112	2.9824	3.8917	3.5705	3.7404	3.5626
3.5629	3.349	3.3006	3.0003	4.0062	3.3852	3.4047	3.5738
3.8035	3.3547	3.6589	2.8524	3.8519	3.473	3.3905	3.6114
3.6267	3.3817	3.5544	2.9614	4.126	3.8271	3.4119	3.5562

without noise

3.6999	3.4229	3.7687	2.926	3.7467	3.7023	3.4117	3.3404

theoretical value

3.6756866

Processing

Trueness (bias)

0.7%	6.9%	2.5%	20.4%	1.9%	0.7%	7.2%	9.1%

Precision error

4%	2%	11%	2%	7%	3%	3%	0%

Real part of the polarizability of the NP [10-3 /µm3]

with noise

DHM	CGM	DPM	DPC	PSI	FPM	SLIM	TIE
-0.8455	-1.6143	-0.5753	-2.5336	-1.4121	-1.0081	-1.1731	-0.0973
-1.2451	-1.262	-1.6733	-2.206	-0.9952	-0.9598	-1.1327	2.456
-0.6803	-1.2529	-1.6844	-1.2751	-0.8103	-1.0509	-1.4698	-0.1593
-1.5247	-0.9172	-1.3405	-1.4881	-1.0792	-1.0316	-1.3739	0.1075
-1.2881	0.0109	-1.8498	-1.1867	-0.6124	-1.09	-1.4758	-0.8445
-1.1921	0.0213	-1.207	-0.9765	-0.9755	-1.2232	-1.0546	-0.4661
-1.2053	-1.3766	-1.0576	-1.6405	-1.2341	-0.9513	-1.4612	-0.8493
-1.2791	-0.5471	-0.9564	-1.6552	-1.5865	-1.0676	-1.3341	-0.2815
-1.0195	-0.6549	-1.0025	-0.8423	-1.1558	-1.0368	-1.3773	-0.82
-1.1808	-0.5572	-1.1349	-2.0457	-1.7073	-0.8089	-1.1093	-3.2866
-1.3854	-1.4295	-0.9471	-1.6648	-0.7607	-0.988	-1.3696	-2.1963
-1.3993	-1.7513	-1.5562	-0.5492	-0.786	-0.9966	-1.3119	-0.7989

without noise

DHM	CGM	DPM	DPC	PSI	FPM	SLIM	TIE
-1.2867	-1.0245	-1.3304	-1.3087	-0.9927	-1.2146	-1.275	-1.195

theoretical value

-1.3037692

Processing

Trueness (bias)

DHM	CGM	DPM	DPC	PSI	FPM	SLIM	TIE
1.3%	21.4%	2.0%	0.4%	23.9%	6.8%	2.2%	8.3%

Precision error

DHM	CGM	DPM	DPC	PSI	FPM	SLIM	TIE
18%	46%	29%	44%	26%	7%	11%	104%

Dry mass of the bacteria [pg]

with noise

DHM	CGM	DPM	DPC	PSI	FPM	SLIM	TIE
0.0959	0.0859	0.0936	0.0951	0.0974	0.0939	0.0832	0.093
0.0934	0.0833	0.091	0.0956	0.0933	0.0948	0.0833	0.1062
0.0934	0.1027	0.0964	0.0937	0.0969	0.0948	0.0852	0.0508
0.0964	0.08	0.0954	0.0973	0.0943	0.0949	0.0831	0.1138
0.0941	0.0888	0.0965	0.0965	0.093	0.0955	0.0825	0.0817
0.0948	0.0951	0.0936	0.1005	0.0937	0.0936	0.0854	0.1045
0.0932	0.0898	0.0936	0.0974	0.0961	0.0959	0.0834	0.0618
0.0955	0.087	0.0937	0.0999	0.0909	0.0951	0.0826	0.0813
0.0957	0.0854	0.0953	0.1034	0.0923	0.0946	0.0845	0.0834
0.0951	0.0895	0.0951	0.0998	0.0966	0.096	0.0834	0.1086
0.0932	0.0977	0.0943	0.0988	0.0927	0.0946	0.0843	0.0664
0.0926	0.0821	0.0942	0.0953	0.1	0.0952	0.0838	0.1323

without noise

0.0949	0.0937	0.0942	0.0984	0.0949	0.0948	0.0837	0.0938

theoretical value

0.094528392

Processing

Trueness (bias)

97.4%	97.4%	97.4%	97.3%	97.4%	97.4%	97.7%	97.4%

Precision error

0%	0%	0%	0%	0%	0%	0%	1%

Dry mass of the neuron [pg]

with noise

DHM	CGM	DPM	DPC	PSI	FPM	SLIM	TIE
74.849	73.174	67.92	46.773	75.331	51.791	26.965	78.481
74.813	73.088	67.903	46.761	75.291	51.783	26.971	80.284
74.815	73.34	67.948	46.662	75.337	51.796	26.973	70.021
74.817	73.218	67.957	46.835	75.383	51.784	26.98	62.819
74.847	73.397	67.951	46.789	75.415	51.789	26.967	68.99
74.835	73.266	67.948	46.61	75.351	51.803	26.973	65.071
74.815	73.216	67.848	46.63	75.323	51.779	26.955	60.769
74.821	73.204	67.913	46.753	75.323	51.785	26.965	77.243
74.86	72.979	67.908	46.676	75.416	51.793	26.961	74.203
74.828	72.971	67.912	46.641	75.403	51.784	26.967	73.318
74.851	72.93	67.969	46.75	75.441	51.806	26.981	77.341
74.814	73.256	67.895	46.796	75.392	51.794	26.977	73.817

without noise

74.827	73.254	67.932	46.723	75.369	51.786	26.971	74.174

theoretical value

75.375763

Processing

Trueness (bias)

######	######	######	######	######	######	633.8%	######

Precision error

0%	4%	1%	2%	1%	0%	0%	174%

OPD of graphene [nm]							
with noise							
**DHM**	**CGM**	**DPM**	**DPC**	**PSI**	**FPM**	**SLIM**	**TIE**
0.4781	0.508	0.4804	0.3632	0.5053	0.4494	0.2674	0.0173
0.4698	0.5873	0.4666	0.381	0.4832	0.4432	0.2647	0.3835
0.5027	0.6564	0.4751	0.3821	0.5046	0.4494	0.2801	-0.6778
0.4712	0.5481	0.4683	0.3637	0.4978	0.4473	0.2833	1.1762
0.4698	0.518	0.4974	0.3517	0.4992	0.4515	0.2684	-0.6175
0.4923	0.4035	0.475	0.3876	0.4955	0.4396	0.2725	-0.0791
0.483	0.5376	0.4693	0.4347	0.4834	0.4531	0.2698	0.3136
0.4826	0.6044	0.4906	0.391	0.4807	0.4423	0.2673	0.3508
0.492	0.5911	0.4737	0.3906	0.4894	0.4451	0.2687	0.9453
0.4733	0.6723	0.4659	0.3921	0.4935	0.4559	0.2611	0.2638
0.486	0.4862	0.4804	0.4244	0.4743	0.4435	0.2696	0.0717
0.4712	0.5708	0.4732	0.3815	0.4774	0.4513	0.2699	0.2317
without noise							
0.4844	0.4848	0.4772	0.3865	0.4847	0.4465	0.2684	0.4846
theoretical value							
0.4848164							
**Processing**							
Trueness (bias)							
86.8%	86.8%	87.0%	89.5%	86.8%	87.9%	92.7%	86.8%
Precision error							
0%	2%	0%	1%	0%	0%	0%	15%
